# 

**Published:** 1907-03-15

**Authors:** 


### ANNOUNCEMENTS.

NATIONAL ASSOCIATION OF DENTAL EXAMINERS.—The National Association of Dental Examiners will hold their twenty-fifth annual meeting in Minneapolis, Minn., beginning Friday, July 26th, and continuing through the 27th and 29th.

Accommodations have been secured in the leading hotel at Minneapolis, "The West Hotel". Rates as follows: Room, without bath, $1.00 per day for each occupant. Room, with bath, $2.00 per day for one person and $1.50 per day for each additional person in room. Hotel on European plan. Any room in the hotel capable of accommodating two people. Telephone in each room, hot and cold water. A large attendance of delegates is earnestly requested. Committee on College, Joint Conference Committee, Tabulation of Examining Boards Reports, The Committee for Promoting a System of Credits and Uniformity of Examinations, will all give exceedingly interesting reports valuable to all the members of the Association. Railroad rates will be announced at a later date. For information apply to Charles A. Meeker, D.D.S., Secretary and Treasurer, 29 Fulton St., Newark, N. J.

BOARD OF DENTAL EXAMINERS OF CALIFORNIA.—At the meeting of the Board of Dental Examiners of California, held in December, 1906, the following officers were elected: President, Garrett Newkirk, Pasadena; Secretary, C. A. Herrick, Jackson; Treasurer, Joseph Loran Pease, Oakland.

The next meeting of the Board will be held in Los Angeles beginning on the second Monday in June. This will be followed by an examination in San Francisco, beginning on the third Monday in June.

C. A. Herrick, Secy., Jackson, Cal.

KENTUCKY STATE ASSOCIATION.—The next meeting will he held at Louisville, May 20th to 22nd 1907. A most cordial invitation is extended to the profession generally. An excellent program is in preparation.

W. M. Randall, *Secy.*

---

NEW YORK STATE SOCIETY.—The Annual meeting will be held at Albany, May 10th to 11th 1907. This Society always has an interesting program and this years will be no exception. Eminent essayists and clinicians.

Chas. S. Butler, *Secy.*,
Buffalo.

---

REMOVAL.—Dr. J. A .Gorman of Asheville, N. C. has removed to Philadelphia, and will practice orthodontia as a specialty at 1623 Walnut Street.

---

OFFICERS CHICAGO ODONTOGRAPHIC SOCIETY.—At the annual meeting the following officers were elected for the ensuing year: President, F. E. Roach; Secretary, F. H. Zinn; Treasurer, G. W. Dittmar.

---

NEW YORK SEVENTH DISTRICT SOCIETY.—The thirty-ninth annual meeting of the Seventh District Dental Society will be held at Colonial Hall, Rochester, N. Y., March 26th and 27th. We expect to have the largest and best meeting in the history of the Society, having secured the assistance of some of the most prominent men in the profession.

From the present out-look, we are going to have a very large exhibit.

Arrangements are being made for reduced railroad rates.

C. W. LaSalle, *Secy.*

